# Small bowel metastasis from endometrial cancer presenting as a bowel obstruction: A case report with literature review

**DOI:** 10.1002/deo2.70117

**Published:** 2025-04-16

**Authors:** Yasuhiko Hamada, Hiroki Yukimoto, Yohei Ikenoyama, Yuhei Umeda, Yasuko Fujiwara, Akina Shigefuku, Hiroto Suzuki, Misaki Nakamura, Noriyuki Horiki, Hayato Nakagawa

**Affiliations:** ^1^ Department of Gastroenterology and Hepatology Mie University Hospital Mie Japan

**Keywords:** balloon enteroscopy, endometrial neoplasm, intestinal obstruction, neoplasm metastasis, small intestine

## Abstract

This report describes a rare case of small bowel metastasis from endometrial cancer, diagnosed six years after initial treatment. A 62‐year‐old woman with a history of grade 2 stage IA endometrial cancer, previously treated with hysterectomy and bilateral salpingo‐oophorectomy, presented with intermittent abdominal pain and nausea. Imaging studies revealed small bowel obstruction and balloon‐assisted enteroscopy identified an annular ulcer with luminal narrowing in the jejunum. Histopathological examination of the biopsy specimen suggested carcinoma; however, its primary origin remained unclear. Subsequent surgical resection confirmed metastatic endometrial adenocarcinoma based on immunohistochemical analysis, which demonstrated positivity for estrogen receptor and paired box gene 8, while CK7, CK20, and CDX2 were negative. Following surgery, the patient experienced symptomatic relief, and no additional metastatic lesions were detected, leading to a conservative follow‐up strategy. This case highlights the diagnostic utility of balloon‐assisted enteroscopy in detecting rare small bowel metastases. Given that such metastases often remain asymptomatic until reaching an advanced stage, early identification is critical. Furthermore, immunohistochemical profiling plays a crucial role in distinguishing metastatic endometrial cancer from other primary small bowel malignancies. Endoscopists should maintain a high index of suspicion for metastatic involvement in patients with a history of endometrial cancer who present with unexplained gastrointestinal symptoms.

## INTRODUCTION

Metastatic tumors of the small bowel are rare and can originate from various primary malignancies, including those of the lung, kidney, skin, and stomach.^1^ These tumors often present with clinical manifestations, such as anemia, gastrointestinal bleeding, obstruction, or perforation. Balloon‐assisted enteroscopy plays a critical role in the diagnosis and surgical planning of metastatic small bowel tumors by enabling histopathological confirmation or precise tumor localization.^1^

Endometrial cancer is the fourth most common malignancy among women worldwide.^2^ While early‐stage disease is associated with a favorable prognosis and a low recurrence rate, late recurrences and metastases have been reported in various locations years after initial treatment.^3^ However, metastasis to the small bowel is rare, and its true incidence remains undetermined due to its rarity and the frequently asymptomatic nature of the disease until advanced stages.^4^

Here, we present a rare case of small bowel metastasis from endometrial cancer, detected by balloon‐assisted enteroscopy six years after the initial surgical treatment.

## CASE REPORT

A 62‐year‐old female patient presented with a 5‐month history of intermittent abdominal pain and nausea, and was referred to our hospital for further evaluation. The patient had a 20‐pack‐year smoking history but had quit 15 years prior. She also had a history of endometrial cancer (endometrioid adenocarcinoma) for which she was treated with a modified radical hysterectomy and bilateral salpingo‐oophorectomy 6 years earlier. The tumor exhibited no myometrial invasion, cervical stromal invasion, lymph node metastasis, or positive peritoneal cytology, and was classified as grade 2, stage IA cancer according to the International Federation of Gynecology and Obstetrics classification.^5^

**FIGURE 1:**
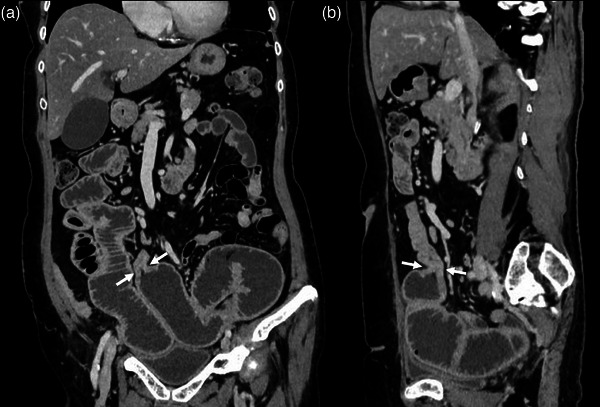
Contrast‐enhanced abdominal computed tomography showing a small bowel wall thickening with proximal loop dilatation (a: coronal image, b: sagittal image, arrows).

**FIGURE 2:**
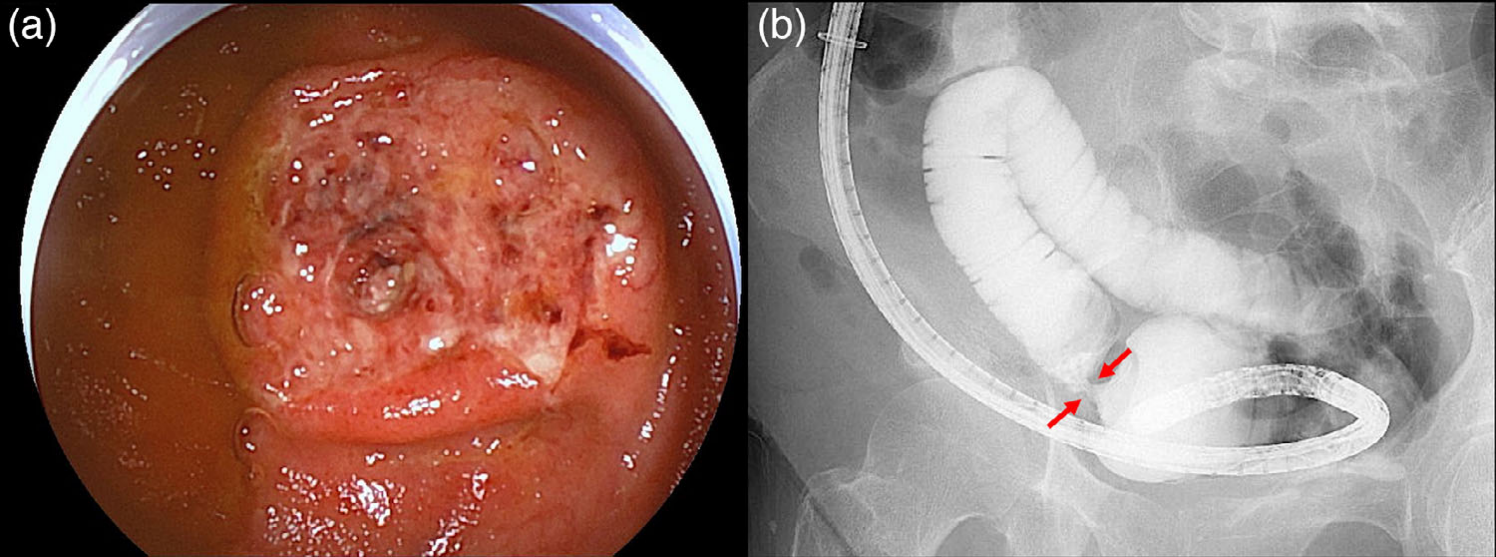
Balloon‐assisted enteroscopy showing an annular ulcer with luminal narrowing in the jejunum (a). Small bowel radiography through the enteroscopy showing an annular stenosis (b, arrows).

**FIGURE 3:**
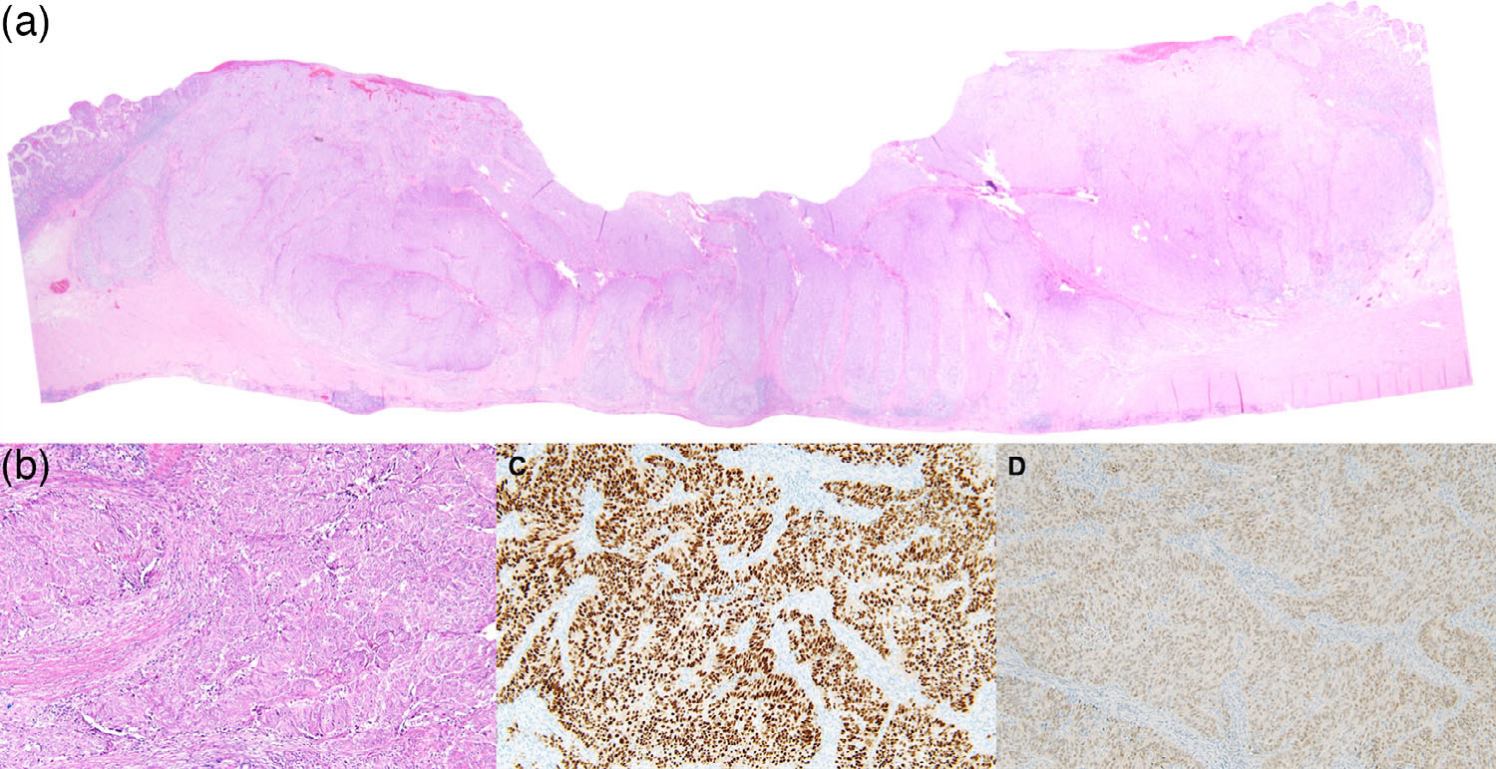
Pathological findings of the resected specimen. Hematoxylin and eosin staining showing that tumor cells are arranged in solid and cribriform patterns, extending from the mucosa to the subserosa (a, original magnification ×12.5, b, original magnification ×100). Immunohistochemical staining (original magnification ×100) showing that the tumor cells were positive for estrogen receptor (c) and paired box gene 8 (d).

On admission, the patient was afebrile, and her vital signs were within normal limits. Physical examination revealed diffuse abdominal tenderness without signs of peritoneal irritation, such as voluntary guarding or rebound tenderness. Laboratory investigations, including complete blood count, liver function tests, renal function tests, and C‐reactive protein levels, were unremarkable. Tumor markers, including CEA, CA19‐9, and CA125, were within the normal range and showed no significant changes over time. Contrast‐enhanced abdominal computed tomography revealed small bowel wall thickening with proximal loop dilatation, indicative of small bowel obstruction (Figure 1a, coronal image; Figure 1b, sagittal image, arrows). No other significant lesions were identified. The patient was initially treated with ileus tube placement. Following the resolution of the bowel obstruction, antegrade balloon‐assisted enteroscopy was performed for diagnostic purposes. The enteroscopy detected a submucosal tumor‐like protruded lesion with luminal narrowing in the jejunum (Figure 2a). Small bowel radiography through the enteroscopy demonstrated an annular stenosis (Figure 2b, arrows). The lesion was biopsied and marked at a site proximal to the lesion by inking. Retrograde balloon‐assisted enteroscopy, in conjunction with small bowel radiography, did not reveal any additional small bowel lesions. Based on the endoscopic findings and imaging studies, the differential diagnosis included malignant lymphoma, metastatic tumors from other organs, and atypical primary small bowel cancer. Initial biopsies of the lesion demonstrated atypical cells that were positive for CK‐AE1/AE3 on immunohistochemical staining, raising suspicion of adenocarcinoma; however, the tissue of origin remained inconclusive. Therefore, a partial resection of the small bowel (proximal jejunum) was performed for diagnostic and therapeutic purposes. Pathological examination of the resected specimen revealed tumor cells arranged in solid and cribriform patterns, extending from the mucosa to the subserosa (Figure 3a,b). Immunohistochemical staining demonstrated that the tumor cells were positive for estrogen receptor (Figure 3c) and paired box gene 8 (Figure 3d) and negative for CK7, CK20, and CDX2. Considering these findings and the patient's history of endometrial cancer, she was ultimately diagnosed with a solitary metastatic small bowel tumor secondary to the endometrial cancer. The patient experienced significant symptom improvement following surgical resection and was discharged 7 days postoperatively. Positron emission tomography/computed tomography revealed no other metastatic lesions. After a thorough discussion, the patient opted for close follow‐up without additional treatment. No recurrence was observed four months after surgical resection.

## DISCUSSION

The most common sites of metastasis or recurrence in endometrial cancer are the pelvic and para‐aortic lymph nodes.^4^ Other typical metastatic sites include the cervix, vagina, peritoneum, and lungs. However, metastasis to the small bowel is extremely rare. The proposed mechanisms of small bowel metastasis secondary to endometrial cancer include: (1) invasion into the small bowel parenchyma via lymphatic metastasis to peri‐small bowel lymph nodes, (2) hematogenous metastasis, (3) peritoneal dissemination, and (4) direct invasion from adjacent organs.^6^ In the present case, no lymph node metastasis was observed in the pelvic or para‐aortic regions at the time of the initial surgery, nor were there findings suggestive of carcinomatous peritonitis during the current surgery. Therefore, hematogenous spread was considered the most likely pathway of metastasis rather than lymphatic or peritoneal dissemination. Regarding the possible hematogenous route, venous drainage from the uterus flows into the internal iliac vein via the venous plexus, suggesting the inferior vena cava as a potential pathway. However, given the rarity of solitary small bowel metastases from endometrial cancer, further case accumulation is necessary for a more comprehensive understanding.

Takahashi et al. analyzed the reported recurrence factors, including older age (>60 years), tumor grade G3, cervical stromal invasion, and positive peritoneal cytology, and developed a novel recurrence prediction score.^7^ Based on this prediction score, the current case exhibited a low recurrence risk. However, reported cases of small bowel metastasis from endometrial cancer included cases with low recurrence risk, warranting careful follow‐up even in these cases.

A systematic MEDLINE search of the English‐language literature up to 2024 using the keywords “endometrial cancer,” “endometrial carcinoma”, “small bowel,” “small intestine,” and “metastasis” identified 13 studies, including the present case, documenting small bowel metastases from endometrial cancer (Table 1; reference articles are listed in Doc S1). The timing of metastasis onset varies considerably, ranging from immediately after the initial surgery to as long as 96 months postoperatively. These metastases are often asymptomatic until the disease reaches an advanced stage and typically present with nonspecific symptoms, such as abdominal pain, anemia, and melena. Consequently, a substantial delay exists from symptom onset to diagnosis and definitive identification.

**TABLE 1:** Reported case of endometrial cancer metastasis to the small intestine (English literature).

Case No.	Author	Year	Patient age (years)	Initial stage	Time period from primary surgery	Clinical symptom	Endoscopy	Metastatic site in the small bowel	Metastatic lesion outside the small bowel	Treatment of small bowel lesion
1	Bosscher et al.	1994	55	IVB	0 month	Not available	No	Proximal ileum	No	Small bowel resection
2	Kirk et al.	1999	64	Not described	10 months	Abdominal pain Melena	No	Mid‐ileum	Spleen	Small bowel resection
3	Thijs et al.	2007	85	IIIB	24 months	Anemia Melena	Capsule endoscopy	Small bowel	Not described	Small bowel resection
4	Tsai et al.	2007	67	Early stage	96 months	Abdominal pain Melena	Upper endoscopy	Duodenum	No	Segmental duodenectomy
5	Gallotta et al.	2015	58	IB	13 months	Not described	No	Small bowel	Vaginal cuff	Small bowel resection
6	Hubers et al.	2017	75	IB	36 months	Hematochezia	No	Proximal jejunum	Sigmoid colon	Small bowel resection
7	Leitão et al.	2017	72	Not described	60 months	Melena	Upper endoscopy	Duodenum	Paraaortic mass	Palliative chemotherapy
8	Makki et al.	2019	88	Not described	5 months	Anemia Abdominal pain	No	Mid‐jejunum	Mesenteric lymph node	Small bowel resection
9	Huynh et al.	2019	Not described	Not described	36 months	Back pain	Upper endoscopy	Duodenum	Retroperitoneal mass	Duodenal resection
10	Singh et al.	2019	77	IA	41 months	Melena Anemia	Upper endoscopy	Duodenum	Retroperitoneal mass	Radiotherapy
11	Emiloju et al.	2020	60	Not described	10 months	Abdominal pain Anemia	Upper endoscopy	Duodenum	No	Pancreatico‐ duodenectomy
12	Nikolvski et al.	2024	88	II	14 months	Cutaneous fistula	No	Mid‐ileum	No	Small bowel resection
13	Present case	2025	62	IA	72 months	Abdominal pain Vomiting	Balloon‐assisted enteroscopy	Distal jejunum	No	Small bowel resection

Reports on endoscopic findings are limited, particularly for lesions distal to the ligament of Treitz. To date, only a single case has been documented using capsule endoscopy, which revealed luminal narrowing, similar to the findings in the present case.^3^ However, metastatic small intestinal tumors exhibit diverse endoscopic features, making definitive diagnosis based solely on endoscopic findings challenging. Therefore, immunohistochemical analysis plays a crucial role in achieving an accurate diagnosis. While primary bowel adenocarcinomas typically express CDX2, CK7, and CK20, endometrial adenocarcinomas are negative for CDX2 and CK20. CK7 is generally positive in endometrial cancer; however, it is negative in approximately 10% of cases, including the present case.^8^ Estrogen receptor and paired box gene 8 are often positive, and notably, paired box gene 8 serves as a useful diagnostic marker for identifying tumors of Müllerian duct origin.^9^

Surgical resection has been the primary treatment modality in previously reported cases, primarily to alleviate symptoms. A previous meta‐analysis showed that complete resection of the tumor is the only significant prognostic factor for improving outcomes in recurrent endometrial cancer.^10^ However, the optimal management following cytoreductive surgery remains inconclusive. A comprehensive approach is required, incorporating radiotherapy, systemic chemotherapy, or observation without further intervention, while considering the patient's health status, metastasis, time interval from primary treatment to recurrence, and initial treatment modality. In the present case, positron emission tomography/computed tomography did not reveal any additional recurrent lesions. Moreover, a meta‐analysis indicated that adjuvant chemotherapy is negatively associated with survival.^10^ Therefore, after a thorough discussion with the patient, we opted for close follow‐up without additional treatment.

In conclusion, endoscopists should maintain a high index of suspicion for metastatic small bowel tumors originating from endometrial cancer to facilitate timely diagnosis and prompt treatments.

## CONFLICT OF INTEREST STATEMENT

None.

## ETHICS STATEMENT

All procedures were performed in accordance with the ethical standards of the Declaration of Helsinki and its later amendments.

## PATIENT CONSENT STATEMENT

Informed consent was obtained from the patient for the publication of this case report.

## Supporting information



Reference articles in Table 1
