# Supplementary material for: Small bowel metastasis from endometrial cancer presenting as a bowel obstruction: A case report with literature review
Source: DEN Open. 2025 Apr 16;5(1):e70117. doi: 10.1002/deo2.70117 (PMC12003207; doi:10.1002/deo2.70117)
Supplement: Supplementary file 1 — Reference articles in Table 1 [file DEO2-5-e70117-s001.docx]

**Reference articles in Table 1**

**Case no. 1**

Bosscher J, Barnhill D, O’Connor D, Park R. Clinical stage IB endometrial adenocarcinoma with an isolated small bowel metastasis. Gynecol Oncol 1994;52:99–101.

**Case no. 2**

Kirk GR, O’Rourke D, Ashe R, Clements WDB. Small bowel intus-susception in metastatic endometrial carcinoma. Ulster Med J 1999;68:110–3.

**Case no. 3**

Thijs WJ, Karrenbeld A, van der Zouwen L, de Haan L. Metastatic endometrial cancer: a rare intestinal localization. Endoscopy 2007;39:E131.

**Case no. 4**

Tsai WC, Loh CH, Lin SH, Tsao YT. The great fortune of misfortune: an unusual cause of gastrointestinal haemorrhage. Dig Liver Dis 2008;40:73.

**Case no. 5**

Gallotta V, Nero C, Callari C, et al. Laparoscopic management of a small bowel recurrence of endometrial cancer. J Minim Invasive Gynecol 2016;23:160

**Case no. 6**

Hubers JA, Soni A. A rare case of endometrial cancer metastatic to the sigmoid colon and small bowel. Case Rep Gastrointest Med 2017;2017:1–3.

**Case no. 7**

Leitão C, Caldeira A, Banhudo A. A rare cause of intestinal bleeding: duodenal metastasis from endometrial cancer. Rev Esp Enferm Dig 2017;109:596.

**Case no. 8**

Makki MM, Alshaikh MO, Saber AR, Eltayeb YH. Small intestine metastasis from endometrial adenocarcinoma: a case report. Dubai Med J 2019;2:117–20.

**Case no. 9**

Huynh KN, Nguyen BD, Wu KJ. Gastrointestinal: Caval tumor thrombus and duodenal metastasis from endometrial carcinoma. J Gastroenterol Hepatol 2019;34:309.

**Case no. 10**

Singh T, Gandhi D, Arora T, Shapiro J. Upper gastrointestinal bleeding due to metastatic endometrial adenocarcinoma. ACG Case Reports J 2019;6:e00138.

**Case no. 11**

Emiloju O, Candelario N, Dourado C. Metastatic clear cell endometrial carcinoma: an unusual cause of a common clinical presentation. BMJ Case Rep 2020;13:e235051–12.

**Case no. 12**

Nikolovski A, Limani N, Ristova Tancheva A,eet al. Small intestine metastasis from endometrial carcinoma initially presented as enterocutaneous fistula: a case report and literature review. J Surg Case Rep. 2024;5:rjae297.

**Case no. 13**

Our case.
